# General Practitioners perspectives on infant telomere length screening after a pregnancy complication: a qualitative analysis

**DOI:** 10.1093/fampra/cmad064

**Published:** 2023-06-01

**Authors:** Carolyn J Puglisi, Joshua McDonough, Tina Bianco-Miotto, Jessica A Grieger

**Affiliations:** School of Agriculture, Food and Wine, University of Adelaide, Adelaide 5005, Australia; School of Public Health, University of Adelaide, Adelaide 5005, Australia; School of Agriculture, Food and Wine, University of Adelaide, Adelaide 5005, Australia; Robinson Research Institute, University of Adelaide, Adelaide 5005, Australia; Robinson Research Institute, University of Adelaide, Adelaide 5005, Australia; Adelaide Medical School, University of Adelaide, Adelaide 5005, Australia

**Keywords:** Australia, GPs, infant screening, pregnancy complication, telomere length

## Abstract

**Background:**

Pregnancy complications can impact the mother and child’s health in the short and longterm resulting in an increased risk of chronic disease later in life. Telomere length is a biomarker of future cardiometabolic diseases and may offer a novel way of identifying offspring most at risk for future chronic diseases.

**Objective(s):**

To qualitatively explore General Practitioners’ (GPs) perspectives on the feasibility and uptake for recommending a telomere screening test in children who were born after a pregnancy complication.

**Methods:**

Twelve semi-structured interviews were conducted with GPs within metropolitan Adelaide, South Australia. Interviews were audio recorded, transcribed verbatim, and analysed for codes and themes.

**Results:**

Two themes were generated: ethical considerations and practical considerations. Ethically, the GP participants discussed barriers including consenting on behalf of a child, parental guilt, and the impact of health insurance, whereas viewing it for health promotion was a facilitator. For practical considerations, barriers included the difficulty in identifying people eligible for screening, maintaining medical communication between service providers, and time and financial constraints, whereas linking screening for telomere length with existing screening would facilitate uptake.

**Conclusions:**

GPs were generally supportive of potential telomere screening in infants, particularly via a saliva test that could be embedded in current antenatal care. However, several challenges, such as lack of knowledge, ethical considerations, and time and financial constraints, need to be overcome before such a test could be implemented into practice.

**Key messages**

- General Practitioners are supportive to include a newborn telomere screening test into postnatal care.
- Barriers include consent, parental guilt, and the impact of health insurance.
- Education programmes and the relevance of follow-up screening in children are needed.

## Introduction

In Australia, around 25% of all pregnancies are complicated by major pregnancy complications such as gestational diabetes mellitus, preeclampsia, pre-term birth, and intrauterine growth restriction,^1^ contributing to increased risk for several cardiometabolic diseases in offspring.^2,3^ Offspring born after a pregnancy complication may be born large or small for gestational age,^4,5^ have increased risk for perinatal morbidity and mortality,^6^ but they are also at increased risk for obesity and insulin resistance,^7^ type 2 diabetes,^8^ and hypertension^9^ in childhood or later life. While not all infants born from a pregnancy complication will have adverse health outcomes, there is a clear need to identify offspring early in life who may be at risk for potential adverse health outcomes in the longer term.

The identification of those most at risk is complex; disease may not manifest until adulthood, but the feasibility of monitoring every child would impose a large strain on healthcare resources. Birthweight is a risk factor for future disease, with a 14% increased risk of cardiovascular disease in adulthood being reported for low birthweight and an 8% increased risk for high birthweight.^10^ However, birthweight has not been shown to produce policy changing evidence for its use as a tool for predicting future disease risk.^11^ A screening tool to identify children who are most at risk would improve long-term health outcomes by identifying individuals that should be monitored for the disease.

Measurement of telomere length offers a novel way of identifying offspring who may be at greater risk of chronic disease. Telomeres are repeating sequences of nucleotides found at the ends of chromosomes, and their main function is to maintain chromosomal integrity.^12^ Several studies in adults demonstrate shorter telomere length is associated with diabetes, cardiovascular disease, and cardiovascular mortality.^13,14^ Pregnancy complications such as gestational diabetes,^15^ preeclampsia,^16^ intrauterine growth restriction,^17^ and even maternal metabolic syndrome,^18^ are associated with shorter telomere length in offspring. Thus, the measurement of telomere length, a biomarker of chronic diseases, has the potential to identify children with a higher risk of future disease.

The role of the General Practitioner (GP) in providing care for all people, including postnatal care is vital in optimising outcomes for families. GPs are accessible and affordable for the majority of the population.^19^ In lieu of a national approach to follow up after a pregnancy complication, screening for telomere length in general practice may be effective, as more than 90% of Australians visit a general practitioner at least once a year.^19^ A previous study amongst health professionals in New South Wales (Australia), highlighted barriers towards child developmental surveillance programmes and screening tools, including time, tool awareness, knowledge, and access to referral pathways.^20^ A systematic review on the acceptability of a range of childhood screening interventions found parents had doubts in the effectiveness of the test or in the accuracy of results (*n* = 6 studies), however, when screening might occur in venues that required minimal effort from the parent, the acceptability was increased (*n* = 3 studies).^21^ Whether telomere length might be useful as a screening tool in newborns born from a pregnancy complication has not been investigated. This study aims to qualitatively explore GPs perspectives on the feasibility and uptake for recommending a telomere screening test in children who were born after a pregnancy complication.

## Methods

### Design

A qualitative design using semi-structured interviews was employed to elicit the views of general practitioners on the usefulness and feasibility of a blood test to measure telomere length in children born of a complicated pregnancy to determine the risk of future chronic disease.

### Participants and recruitment

GPs within South Australia were purposively recruited; (i) through practice managers by email communication and (ii) through collaboration with a not-for-profit organisation, specialising in GP and GP Obstetrician education and support. Our research was presented at education sessions and expressions of interest registered. Eligibility criteria included GPs who were currently practicing in South Australia and had practiced for at least one year in South Australia and could communicate fluently in English. Recruitment took place between November 2021 and March 2022. Initially, 19 general practices were contacted by phone, and information packages were sent by email to practice managers. Emails were followed up with phone communication to ensure information was received and distributed to GPs. Recruitment was slow in the first 2–3 months, however, this was during a recent surge of the COVID-19 pandemic in Australia. Only four GPs were recruited through practice managers. We then focussed on snowballing recruitment via GP Partners, a not-for-profit organisation specialising in shared care education and training of GPs and GP-obstetricians. Two PowerPoint presentations at two GP Partners’ education sessions were delivered to approximately 50 GPs to explain the study and establish a rapport with potential interviewees. GPs were offered a $50 gift voucher to participate. In total, 14 GPs expressed interest [10 who were affiliates of GP Partners and 4 from smaller GP clinics], and all met study eligibility. Two GPs declined an interview due to personal reasons or time restrictions. All GPs consented to have their interview audio-recorded, except one, for personal reasons, but a thorough, verbatim, written transcript was collected and included in the analysis. All interviewees provided verbal consent. Ethical approval was granted by The University of Adelaide Human Research Ethics Committee [H-2021-110, date approved 2021 October 26].

### Data collection

This study was guided by the Consolidated criteria for reporting qualitative studies (COREQ),^22^ to enhance research validity (purposeful sampling, audit trail), rigour (data saturation, ethics approval), credibility (member checking), and generality (inclusion criteria). Interviews were semi-structured based on the study objectives. Each GP described their knowledge regarding pregnancy complications and any relevant follow-up guidelines/recommendations; attitudes regarding the potential implementation of a screening test in children; facilitators and barriers regarding potential usage and uptake. The interviews utilised a reflective approach^23^ allowing flexibility for the researcher to either elaborate or clarify certain responses without a pre-established assumption. The interview was facilitated by an interview schedule with open-ended questions designed to allow participants to respond, while also elaborating on areas they considered to be important. Several guiding questions were created to ensure that the interview generated relevant data. Basic demographic data, including age, gender, years of practice in medicine/GP, and location (metropolitan/rural) was collected from each participant. Prior to commencement, the questions were pilot tested on two researchers and two GPs, with slight modifications made to improve clarity. The semi-structured telephone interviews were conducted by one researcher (CP), following training by an experienced qualitative researcher (JM). CP is a research scientist with experience in acute care (intensive care and high dependency) hospital ward work (both surgical and medical), and interview conduct. JM is a PhD graduate with expertise in qualitative data analysis, and qualitative research in the GP setting. Third author, TBM, is a senior research scientist with expertise in epigenetics (including telomere length). The last author, JAG, is a mid-career research scientist with experience in qualitative data collection and analysis, and telomere length biology. A single interviewer allowed for continual and simultaneous data collection, cross-checking of themes, and confirmation of data saturation. No interviews were repeated. GPs were given the opportunity to review the transcripts but provided no further comments.

### Data analysis

De-identified audio recordings were reviewed by CP [a trained research fellow] for accuracy and were transcribed using a professional transcription service (Pacific Transcription). Transcripts were thematically analysed and coded using NVivo version 12 Plus (Windows) 2018 QSR International Pty Ltd Software. Using the Braun and Clarke method,^24^ familiarising the transcript is the first step in data analysis, followed by the identification of elements of interest in the data. These initial codes were then linked together to create themes. Final themes were reviewed to ensure they were reflective of the original transcript and the research question. JM coded all transcripts, CP independently coded a third of the transcripts, which were then reviewed and compared with coding by JM to ensure consistency and reliability. Discussions between investigators were conducted three times to gain a consensus on codes and themes.

## Results

From the 33 practice managers contacted, 12 GPs participated in the phone interviews, 11 of whom were female. The GP age range was 33–70 years, all were from the metropolitan area, and the interview time was around 24 min. Two themes were generated from the data: ethical considerations and practical considerations. Ethically, the GP participants discussed barriers including consenting on behalf of a child, parental guilt, and the impact of health insurance, whereas viewing it for health promotion was a facilitator. For practical considerations, barriers included the difficulty in identifying people eligible for screening, maintaining medical communication between service providers, and time and financial constraints, whereas linking screening for telomere length with existing screening and vaccination schedules in South Australia could help increase the uptake of screening while limiting impact on general practice resources.

## Ethical considerations

### Consenting on behalf of a child

GP participants discussed that there was no guarantee that, should the screening be performed during infancy, that these patients would want to know the results or have consented to the screening later in life. This was contrasted to the childhood immunisation schedule, where the benefits are clearer and less likely to have negative outcomes in adulthood, compared to telomere length screening where the evidence is uncertain.

I guess there’s some other ethical things involved in the parents consenting for [screening] but the child might not want them to have known or wanted it out in that sort of public domain, before they were old enough to consent. GP08

The tricky part with this is these are decisions being made by parents on behalf of their children before their children can actually consent. When you’re getting to 18/19/20 and you’ve been presented with this information that you maybe didn’t want to know in the first place, that’s another element to navigate. GP04

### Parental guilt

Participants noted that it may be difficult to manage the potential guilt of parents caused by the results of the screening. As complications in pregnancy (e.g. preeclampsia) can be a result of stigmatised lifestyle factors (e.g. obesity), it may be that a negative outcome from the screening could lead mothers to feel a sense of guilt and responsibility for having potentially contributed poorer health outcomes for their child. Distress to the parent may reduce the net benefit of screening for shortened telomere length in the child.

Guilt is also a concern, as many patients seem to suffer from some kind of guilt during those early years of parenting, this will add to an already existing degree of guilt. GP07

Potentially whether the parent will feel guilt if they do find out … or [if it] will happen in the future. GP11

I would feel worried that the mum would be made to feel guilty for the pregnancy being a potential cause or risk factor for this child having chronic illness in the future, and often those complications are actually not within the mum’s control. GP12

### Health insurance

Many participants contemplated what the results of screening for telomere length could have on health insurance within Australia. There was concern that tests identifying shortened telomere length, and therefore higher risks of chronic disease, could be used as justification for health insurance companies to increase costs, or refuse coverage for certain conditions. Participants noted that safeguards would need to be in place to protect the interests of patients.

When you’re looking at telomere length and its association with chronic disease maybe, and degenerative diseases later on in life, are the insurers going to grab hold of it – we really don’t know. GP01

Insurance may be an issue- will insurance companies insure if a positive test is obtained? GP07

### Health promotion

Participants noted that it is important to be able to provide information to people to maximise their ability to make health-altering decisions. This spoke to the role of general practice and primary care, being preventative medicine and monitoring chronic conditions. They noted that some of their patients have an appetite for information that could benefit their health and who would be interested in knowing any risk factors their child may carry. However, this was contrasted with the question of how a patient would be able to action the results of the screening, and the potential connotation that it may relate to genetic screening.

I think the more prevention the better, and the more awareness, the more that we are aware that these things could lead on, it would be fabulous. GP01

It’s actually a health promotional tool, I think it’s actually very good. However, in the hands of the right people it’s actually got a lot of danger. GP06

I think if we can pick up children who are at risk and perhaps, we can intervene…providing the education, via providing referrals through a multi-system, multidisciplinary team…we can treat these children and monitoring them better regularly. GP11

What’s the point of screening for something, in some people’s minds, if you can’t do anything about it? Is there evidence to say that, yes, you can do stuff to reverse it, minimise it? GP03

## Practical considerations

### Identifying infants eligible for screening

GP participants mentioned that they would find it difficult to identify patients who would be eligible for telomere length screening. Those, in general, practice, unless involved in perinatal care, would be unaware of the conditions of the pregnancy, and therefore less likely to mention it in consults and be more dependent on notes from the obstetrician. This was partly acknowledged as a result of this being an emerging area of research, as well as the difference in skills and interest levels of general practitioners. It was stated that only those with experience in maternal health, and a keen interest in current research would be aware of the impact of telomere length, and the potential to screen for those at risk of negative health outcomes. Furthermore, the indication that there appeared unclear guidelines regarding the follow up of children born after a pregnancy complication was a barrier to identifying and treating a child born after a pregnancy complication.

…hopefully that information would be recorded in our patient record. It would often

be lost, I think … not necessarily really visible, especially as the child gets older. GP10

Yeah, I think there’s always a barrier for the GPs knowing about it, spreading the word to the GPs that don’t do the antenatal care, don’t keep up to date on these particular things. GP03

No idea if there’s guidelines around but any child that’s born either premature or a significant complication then basically, I tend to see them at their regular immunisations but also six, nine and then again at 12 months. GP06

### Communication between doctors

Participants noted that maintaining streams of communication between treating doctors could be an issue in screening for shortened telomeres. Given that patients may move and/or change between practices, it would be difficult to have the results of the screening follow parents and their children. This barrier was further complicated when considering the timing of the screening. When contemplating screening in infancy, participants noted the custodian of the results to be an important consideration, as the baby is unable to hold the information, and not guaranteed to see the general practitioner who organised the test.

Well, if I had a discharge summary it might be included on that if it’s been given to me in a timely manner. I received one last week from six months ago. GP09

We can access My Health Record so we get an idea of what the discharge summary would have said... beyond that it’s reliant on history checking. GP05

We’d rely heavily on the obstetrician and on letters. GP08

### Time and financial constraints

Almost all participants noted the time limitations within general practice. This was noted in the difference in appointment styles, with some general practitioners valuing longer appointment times, and others providing shorter, bulk-billed appointments. With current limitations, particularly in regional and remote areas, wait lists for non-urgent appointments were noted to be up to 10 weeks.

Absolutely time constraints, that’s always an issue in GP Land. That would always be a challenge. GP04

I guess the usual barriers like time management, that’s the general one that applies to everywhere not just screening tests, everything we do are always time limited. GP11

Along with time constraints, participants noted that there would need to be financial incentive for screening to be widespread. Some noted that current consultations for antenatal care are poorly remunerated, which is a disincentive for general practitioners to devote more of their time to these issues. Additionally, were the screening to not be a Medicare rebate for doctors, then this cost would be relayed to the parent, which may create equity issues.

Doing those things is time consuming and poorly remunerated but you can say that for all of general practice. GP09

I wonder whether financial costs whether it comes out of the cost to the patient, if it does then, some of them may not want to have it. GP11

### Integrating telomere length screening with existing screening

Facilitators included the linking of screening for telomere length to existing appointments in antenatal care. Other appointments where information is provided (such as antenatal appointments) and a health-protective procedure occurs could save time and increase convenience for both general practitioners and parents, increasing the likelihood of screening. In Australia, the newborn screening test (heel prick to screen for cystic fibrosis, amongst other genetic conditions) and the childhood immunisation schedule (i.e., vaccines given at two, four, and six months), were identified as timepoints in antenatal care within Australia, where telomere length screening could be integrated. The use of a blood test appeared suitable if integrated into current tests, however, the option of a saliva sample would facilitate its use.

When a baby’s born, they have the heel prick test for screening of cystic fibrosis and things like that … Could it be done in hospital before they go as one more test? GP03

These conversations would be happening at the routine immunisation schedule visit … they could maybe be doing [telomere length screening]. GP02

I guess the easier it is to do the test, like if I can take it in my room with a swab of saliva, great. If I have to send them off to get a blood test, less great. GP09

Nowadays I don’t think many people will be opposed to having a blood sample done as long as we explain how we’re going to do it, and how we can apply anaesthetic cream for the blood taking. The saliva sample doesn’t sound too hard to collect as well. GP11

## Discussion

This study explored GPs’ perspectives on the potential implementation of telomere length as a neonatal screening test in Australian general practice. Two themes were generated: ethical considerations, including those surrounding parental consent on behalf of the child, parental guilt, the impact to health insurance, but also the impact for health promotion. Practical considerations encompassed the identification of eligible patients, communication, time, and financial constraints, and the integration of screening into current care.

To our knowledge, no other studies have considered telomere length as a screening tool for future chronic diseases in infants, nor have they qualitatively explored the physician’s attitudes regarding its potential use. However, several studies in adults have frequently measured telomere length in blood, demonstrating shorter telomeres are associated with increased risk of cardiometabolic disorders.^13,14^ In children, there have been less studies investigating telomere length; however, those that have, consistently show that shorter telomeres are associated with adverse events early in childhood.^25,26^ If a test that measures telomere length in newborns could identify offspring at increased risk of future chronic disease, before disease is apparent in adults, this would have a critical impact on breaking the cycle of future chronic disease risk. Our findings add significant novelty to current literature that has suggested a proposed use, by exploring attitudes and barriers towards its potential uptake as a screening tool.

Critical barriers towards telomere length screening were also raised, with consenting on behalf of the child an important ethical consideration. GPs in the current study were reflective of when offspring at an older age may not have approved of their parents consenting to the newborn screening on their behalf. Since the 1960’s, healthcare providers in Australia have offered newborn bloodspot screening to babies in all states and territories. Consent is voluntary and is grounded in a range of ethical concepts, as highlighted in the National Policy Framework, and describes recommendations to protect the privacy of the individual from whom the bloodspot was taken.^27^ Universally, newborn screening is recognised as one of the most efficient and effective screening programmes.^28^ However, with increasing opportunities to screen for disorders that previously were difficult to identify in the newborn, this has inherently raised concerns, including those of consent. Specific to genetic testing, consent is based on the benefits for testing outweighing harm and is undoubtedly acceptable. As an example, for Duchenne Muscular Dystrophy, the availability of genomic data requires families to make decisions about information that may predict future events, with differing levels of certainly and the ability to intervene.^29^ Whereas a disease such as diabetes is attributed to both genetics and lifestyle, individual information about personal risk of developing diabetes in the future may not be appropriate for newborn screening.^28^ The concerns of GPs are valid, although telomere length is a biomarker of future disease risk and is not a genetic test. The difficulty is balancing the challenges and benefits of screening. This may involve tailored education to GPs on the role of telomere length as a biomarker and the benefits of early screening, but also education and counselling for parents during the pre- and postnatal period.

A novel, though less surprising aspect to this study was the issue of parental guilt. It was raised by the GPs that many parents already feel guilty in the early years of parenting, and so results of such a screening test may pose an additional burden. Moreover, because pregnancy complications may be in-part, attributable to lifestyle factors such as suboptimal maternal nutrition and physical activity, the potential indication of an increased risk for cardiometabolic diseases in later life would further add to parental guilt. The stigma surrounding such a screening test because of potential parental behaviour is unique to our study, though stigma in reproductive health is an emerging concept in the literature.^30,31^ The developmental origins of health and disease, whereby exposures during early life, including *in utero*, influence the risk of later conditions, creates a potential caveat for where individual responsibility lies. Specifically, because maternal behaviours play a significant role in offspring health outcomes, there is potential risk for ongoing maternal blame for adverse offspring health. Most research to date has focussed on weight stigma across pregnancy and antenatal care, revealing associations with psychological distress, decreased access to and uptake of reproductive healthcare, poorer health behaviours, and adverse pregnancy outcomes.^31^ A recent scoping review on 44 studies on stigma related to gestational diabetes, described the feelings of responsibility and guilt were typically related to concerns for the unborn child, and feeling like they failed the unborn child.^32^ Supportive communication between the doctor–patient and continuity of care may facilitate discussions around emotional experiences and guilt.

Study findings contribute new knowledge by GPs expressing difficulty in identifying infants who might be eligible for screening, specifically those born following a pregnancy complication. It was described that not all GPs are equipped with the knowledge or speciality in maternal health to actively engage with parents/families about the consequences of a complicated pregnancy on the long-term health of offspring. Such knowledge deficit is not an uncommon theme, as GPs see themselves as generalists and are therefore reluctant to initiate testing considered more specialist.^33^ Roth et al.^34^ identified gaps in healthcare provider knowledge regarding maternal cardiovascular risk after hypertensive disorders during pregnancy, with obstetricians tending to have higher rates of understanding of the risks, but still overall knowledge was lacking.^34^ Other studies internationally also reveal knowledge gaps among general practitioners in the assessment, management, or diagnosis of women who have perinatal depression^35^ or vulnerability in pregnancy,^36^ but even a lack of knowledge in terms of child allergies^37^ and child obesity.^38^ Our findings add to the complexity of follow-up screening in both women and their children, where currently, no international consensus for post-partum follow-up care following several pregnancy complications exist,^39^ or where they do, post-partum follow up is low.^40^ These consistencies highlight the ongoing barriers that GPs regularly face, but strengthen the need for education programs, particularly that are compatible and that have reach, for GPs working in pregnancy and postnatal care, and the relevance of follow-up screening in children.

### Strengths and limitations

This study provides an in-depth analysis of the perspectives of GPs to potentially implement a screening test to identify offspring at high risk of future chronic disease. Study findings contribute to the limited knowledge assessing follow-up of screening after pregnancy within Australia and provide novel findings on a potential new screening tool that could be considered early in life. Strengths of this study include the diverse age range and years of GP training, and while all GPs were located within the Adelaide metropolitan area, some of the GPs also had rural experience, enhancing the generalisability of findings. Further research from an international setting would contribute to our findings, along with the potential screening in offspring of culturally and linguistically diverse backgrounds where knowledge and communication gaps are likely larger. There is the possibility that our findings were influenced by desirability bias, where highly motivated and interested GPs were more likely to participate. The views of other health professionals and those of women who had a pregnancy complication may add value to our findings. Finally, despite a relatively small sample size, it was sufficient for data saturation with clear themes generated.

### Conclusions

GPs were generally supportive of potential telomere screening in infants, particularly if aligned with current care. However, critical barriers such as consent, the identification of eligible infants, and the potential guilt that women may face, need to be explored and controlled. Our results highlight the need for further exploration of the potential appropriateness of telomere screening among other health professionals, but also parents, and to further investigate how best such a screening test could be implemented into routine care. It also reiterates the need for transparent follow-up guidelines.

## Supplementary Material

cmad064_suppl_Supplementary_Checklist

## Data Availability

The data underlying this article cannot be shared publicly due to the privacy of individuals that participated in the study.
